# An S-(Hydroxymethyl)Glutathione Dehydrogenase Is Involved in Conidiation and Full Virulence in the Rice Blast Fungus *Magnaporthe oryzae*

**DOI:** 10.1371/journal.pone.0120627

**Published:** 2015-03-20

**Authors:** Zhen Zhang, Jiaoyu Wang, Rongyao Chai, Haiping Qiu, Hua Jiang, Xueqin Mao, Yanli Wang, Fengquan Liu, Guochang Sun

**Affiliations:** 1 Department of Plant Pathology, College of Plant Protection, Nanjing Agricultural University, Nanjing, China; 2 State Key Laboratory Breeding Base for Zhejiang Sustainable Pest and Disease Control, Institute of Plant Protection and Microbiology, Zhejiang Academy of Agricultural Sciences, Hangzhou, China; Soonchunhyang University, KOREA, REPUBLIC OF

## Abstract

*Magnaporthe oryzae* is a hemibiotrophic fungal pathogen that causes rice blast disease. A compatible interaction requires overcoming plant defense responses to initiate colonization during the early infection process. Nitric oxide (NO) plays important roles in defense responses during host-pathogen interactions. Microbes generally protect themselves against NO-induced damage by using enzymes. Here, we characterized an S-(hydroxymethyl)- glutathione dehydrogenase gene in *M*. *oryzae*, *MoSFA1*, the homologs of which are involved in NO metabolism by specifically catalyzing the reduction of S-nitrosoglutathione (GSNO) in yeasts and plants. As expected from the activities of S-(hydroxymethyl)glutathione dehydrogenase in formaldehyde detoxification and GSNO reduction, *MoSFA1* deletion mutants were lethal in formaldehyde containing medium, sensitive to exogenous NO and exhibited a higher level of S-nitrosothiols (SNOs) than that of the wild type. Notably, the mutants showed severe reduction of conidiation and appressoria turgor pressure, as well as significantly attenuated the virulence on rice cultivar CO-39. However, the virulence of *MoSFA1* deletion mutants on wounded rice leaf was not affected. An infection assay on barley leaf further revealed that *MoSFA1* deletion mutants exhibited a lower infection rate, and growth of infectious hyphae of the mutants was retarded not only in primary infected cells but also in expansion from cell to cell. Furthermore, barley leaf cell infected by *MoSFA1* deletion mutants exhibited a stronger accumulation of H_2_O_2_ at 24 and 36 hpi. *MoSFA1* deletion mutants displayed hypersensitivity to different oxidants, reduced activities of superoxide dismutases and peroxidases, and lower glutathione content in cells, compared with the wild type. These results imply that *MoSFA1*-mediated NO metabolism is important in redox homeostasis in response to development and host infection of *M*. *oryzae*. Taken together, this work identifies that *MoSFA1* is required for conidiation and contributes to virulence in the penetration and biotrophic phases in *M*. *oryzae*.

## Introduction

*Magnaporthe oryzae* is a hemibiotrophic fungal pathogen that causes rice (*Oryza sativa*) blast disease. It colonizes plants asymptomatically as a biotroph in susceptible plants before entering its destructive necrotrophic phase [1]. Nitric oxide (NO) produced by the host plays important roles during the plant defense responses [2–4]. Rapid accumulation of NO is induced by recognition of effectors or pathogen-associated molecular patterns (PAMPs), which consequently triggers plant defense response by means of hypersensitive response (HR), phytoalexin biosynthesis and defense gene activation [2, 5–7]. Moreover, NO also has directly antimicrobial activity via cellular damage [8–11]. The excessive NO administration to fungal cells can inhibit the antioxidant enzymes activities, reduce ATP synthesis and increase carbonylation damage [9, 12–13]. The burst of NO in the infected tissues creates a potent antimicrobial environment that conduces to the restriction of the pathogen growth, similar with the burst of reactive oxygen species (ROS). A compatible interaction between plant and pathogen requires overcoming plant defense responses to initiate colonization during the early infection process. However, the means by which pathogens deal with NO is not well understood.

Microbes generally protect themselves against NO-induced damage by using enzymes that convert NO to less toxic molecules. Evidences elaborated that an S-(hydroxymethyl)glutathione dehydrogenase (EC 1.1.1.284), belongs to a class III alcohol dehydrogenase and functions in the glutathione-dependent oxidation of formaldehyde, is involved in NO metabolism though specifically catalyzing the reduction of S-nitrosoglutathione (GSNO) [14], which is the reaction product of glutathione (GSH) and NO, and serves as a naturally occurring mobile reservoir of NO bioactivity [15]. This enzyme is conserved from bacteria to humans, which reduces GSNO to ammonia (NH_3_) and glutathione disulphide (GSSG) in the presence of NADH and GSH. Deletion of its homologs in several species results in increased hypersensitivity to NO and increased accumulation of cellular S-nitrosothiols (SNOs), subsequently causes nitrosative stress [16–17]. Notable, it is well documented in several human pathogens that this enzyme is required to counteract pathogen-induced NO activity and promote virulence. Expression of *adhC* gene in bacterial pathogen *Streptococcus pneumoniae* was strongly induced by GSNO, and *adhC* deletion mutants exhibited hypersusceptibility to NO stress and decreased the fitness in blood [18]. In *Cryptococcus neoformans*, although *GNO1* deletion mutants proliferated normally in vitro with nitrosative challenge and did not reduce the virulence in vivo, *GNO1* can partly promote survival of flavohemoglobin-null mutants in the infected host, which contribute to NO-consuming activity in *C*. *neoformans* [19]. Thus, *GNO1*-mediated GSNO reducing pathway also contribute to virulence of cryptococcal disease. As mentioned above, although NO burst in plants plays crucial roles in defense response, little is known about the roles of S-(hydroxymethyl)glutathione dehydrogenase in plant pathogenic fungi.

In present study, we investigated the function of a S-(hydroxymethyl)- glutathione dehydrogenase gene in *M*. *oryzae*, namely *MoSFA1*, a homolog of *Saccharomyces cerevisiae SFA1*. Our results showed that *MoSFA1* is essential for normal vegetative growth, conidiation and full virulence in *M*. *oryzae*. This information provides initial insights into the physiological and biological functions of *MoSFA1*-associated NO metabolism in the rice blast fungus.

## Material and Methods

### Fungal strains and growth conditions

The wild type *M*. *oryzae* strain Guy11 and its derivative strains were grown on complete medium (CM) plates [20] at 28°C. Conidia were harvested from 9-day-old cultures grown on CM plates. Genomic DNA and total RNA were extracted from 3-day-old cultures grown in liquid CM. Fungal transformants were screened in CM plates supplemented with 200 μg ml^−1^ of hygromycin B (Roche Applied Science, Mannheim, Germany) or 250 μg ml^−1^ of glufosinate ammonium (Sigma-Aldrich, St Louis, MO, USA), depending on the selection marker. Evaluations of conidiation and vegetative growth were performed as previously described [21]. And growth rate of *M*. *oryzae* was determined by measuring the colony diameter of 6-day-old cultures on CM plates supplemented with chemicals.

### Complementation of *Saccharomyces cerevisiae Δsfa1* mutant

The total RNA from *M*. *oryzae* was extracted with TRIzol reagent (Invitrogen, Carlsbad, CA, USA) and DNA traces were cleaned up with Turbo DNAfree kit (Ambion, Austin, TX, USA). The CDS of *MoSFA1* was produced by RT-PCR using a gene-specific primer set MG125/MG126, which was incorporated in the *Hin*dIII and *Xba*I restriction endonuclease sites, respectively. The PCR product was cloned into the pEASY-T3 vector (Transgen Biotech, Beijing, China) and verified by DNA sequencing. The correct plasmid was digested by *Hin*dIII and *Xba*I, and the fragment was ligated into the pYES2 yeast expression vector (Invitrogen, Carlsbad, CA, USA) to generate pYES2-MoSFA1. After sequencing verification, the pYES2-MoSFA1 plasmid was introduced into the *S*. *cerevisiae Δsfa1* mutant strain YDL168W (BY4741:*Mata/his3-1/leu2-0/met15-0/ura3-0/YDL168W*::*kanMX4*) purchased from Thermo Fisher Scientific (Waltham, MA, USA) using the lithium acetate method. For formaldehyde sensitivity assay, yeast cells were incubated in liquid YPD medium (2% glucose, 2% peptone, and 1% yeast extract) with shaking at 28°C for overnight. Cells were collected and washed three times with sterile distilled water. Cell suspensions adjusted to OD_600_ = 1 were 10-fold serially diluted, and aliquots (5 μl) of 10-fold serial dilution were grown in SC medium (0.67% nitrogen base, 2% raffinose, 2% galactose, 2% agar,) containing 1.1 mM formaldehyde at 28°C for 2 days. Primers used in this study were listed in S1 Table.

### GSNO reductase activity assay

GSNO reductase activity was measured spectrophotometrically at 340 nm by the time-dependent oxidation of NADH and reduction of GSNO, as described by Sakamoto *et al*. [22]. Yeast cells were harvested from the cultures grown in liquid YPD medium to OD_600_ 0.5–1.0 and washed three times with sterile distilled water. The cells were resuspended in 150 μl extract buffer [50 mM Tris-HCl, pH 8.0, and 0.1% (v/v) Tween 20], and mechanically broken with acid-washed glass beads in Fastprep-24 Instrument (MP Biomedicals, Solon, OH, USA). Then samples were centrifuged at 4°C for 10 min at 12,000 x g to remove the beads and insoluble material. The concentration of protein extracts was determined by a Bradford assay, with bovine serum albumin (BSA) as the standard. About 10 μg proteins were incubated in 100 μl assay buffer that contains 20 mM Tris-HCl (pH 8.0), 0.2 mM NADH and 0.5 mM EDTA. The reaction was started through adding GSNO (Cayman Chemical, Ann Arbor, MI, USA) at a final concentration of 400 μM. The absorbance was measured at 340 nm at room temperature using a Thermo Varioskan Flash spectrophotometer (Thermo Fisher Scientific, Waltham, MA, USA). The resultant GSNO reductase activity was expressed as nmol NADH consumed min^−1^ (mg protein)^−1^.

### Targeted gene replacement of *MoSFA1* and complementation

In order to replace *MoSFA1* gene, a 1.6-kb upstream flanking sequence fragment and a 1.5-kb downstream flanking sequence were amplified from the genomic DNA of the wild type strain Guy11, with the primer sets MG121/MG122 and MG123/MG124, in which *Bst*XI/*Eco*RI and *Xba*I/*Sal*I restriction endonuclease sites were incorporated, respectively. The PCR products were cloned into pEASY-T3 and verified by DNA sequencing. After digesting the correct plasmids with *Bst*XI/*Eco*RI and *Xba*I/*Sal*I restriction endonuclease combinations, the upstream and downstream fragments were isolated and orderly inserted in the corresponding sites of p1300-KO [23] to generate the *MoSFA1* gene replacement vector pKO-MoSFA1. *Agrobacterium tumefaciens* strain AGL1 was used to transform *M*. *oryzae* as described by Wang *et al*. [21].

Candidate mutants were screened by PCR with the primer sets and gene deletion mutants were confirmed by Southern blotting. The genomic DNA of candidate mutants and Guy11 were digested by *Bst*XI and blotted on Nylon^+^ membrane (Roche Applied Science, Mannheim, Germany) and hybridized with a 1.5-kb fragment as the probe, which was amplified from the 3’-end of the *MoSFA1* gene with the primer set MG129/MG130 and labeled with the DIG High Prime DNA labeling and detection kit II according to the manufacturer's instructions (Roche Applied Science, Mannheim, Germany).

For complementation of the *MoSFA1* deletion mutant, the 3.4-kb fragment containing about 1.8-kb upstream sequence from the start codon ATG, the *MoSFA1* gene coding region, and 0.4-kb downstream sequence from the stop codon TAA was amplified with the primers set MG127/MG128, in which *Eco*RI/*Xba*I restriction sites were incorporated. This PCR product was cloned into pEASY-T3 and verified by DNA sequencing. The correct plasmid was digested by *EcoR*I and *Xba*I, and the fragment was cloned into *Eco*RI/*Xba*I-digested p1300BAR, which carried a glufosinate ammonium resistance marker [23]. The resultant plasmid pBAR-MoSFA1R was verified by sequencing and used to transform *MoSFA1* deletion mutant by *A*. *tumefaciens*-mediated transformation, and fungal transformants were screened on the CM plates supplemented with 250 μg ml^-1^ glufosinate ammonium.

### Pathogenicity assays

For pathogenicity assays, 4-week-old seedlings of rice (*Oryzae sativa* cv. CO-39) were infected with conidia suspensions prepared in 0.25% gelatin at a concentration of 5×10^4^ conidia ml^−1^ using an artist's airbrush with high-pressure air. Inoculated plants were placed in a moist chamber at 25°C for 24 hours in the dark. One day after inoculation, rice seedlings were maintained in the moist chamber with a photoperiod of 12 hours under fluorescent lights for additional 5 days to allow the full development of disease symptoms. Disease severity was scaled as described by Fang and Dean [24] to evaluate the virulence of all tested strains. For wounded inoculation, 20-μl aliquots of serial dilutions of conidia suspension were dropped on rice leaf segments wounded with a pin. The experiments were repeated at least three times with triple replications that yielded similar results.

### Analysis of infection-related morphogenesis

Conidia harvested from 9-day-old CM plates were suspended in sterile water at 5×10^4^ conidia ml^−1^. Aliquots (20 μl) of the suspensions were incubated on a plastic coverslip (Thermo Fisher Scientific, Waltham, MA, USA) at 28°C. Conidial germination and appressorium formation were examined at 48 hours post-incubation (hpi). Appressorium turgor pressure was determined using a cytorrhysis assay [25]. Appressoria were allowed to form on a plastic coverslip at 48 hpi, water was then carefully replaced with 20 μl 1–3 M glycerol solution. After 10 min at room temperature, the number of collapsed appressoria was counted under a light microscope. The experiments were replicated three times, and >200 appressoria were observed for each strain. Penetration and infectious hypha growth were assayed in the barley (*Hordeum vulgare* cv. Golden promise) leaf as described previously [26]. Infected leaves were observed under a light microscope at 36 and 48 hours after inoculation. The experiments were replicated three times, and >50 appressoria were observed for each tested strain.

### Detection H_2_O_2_ in infected host cells

To observe the accumulation of H_2_O_2_ at infected cells, the detached barley leaves were inoculated with conidia suspension at a concentration of 5×10^4^ conidia ml^−1^ using an artist's airbrush with high-pressure air. The infected barley leaves were stained with 3, 3’-diaminobenzidine (DAB, Sigma-Aldrich, St Louis, MO, USA) as described previously by Daudi et al. [27]. Barley leaf segments were incubated in 1 mg/ml DAB solution in the dark at room temperature for 8 hours and decolorized in boiling ethanol (96%) for 10 min. Samples were observed under a light microscope.

### Quantitative RT-PCR

Total RNA (500 ng) was used for reverse transcription and the products were used for the relative gene expression analysis. Quantitative real-time PCR was performed on a 7500 Real-Time PCR System (Applied Biosystems, Foster City, CA, USA) using SYBR Premix Ex Taq kits (Takara, Dalian, China). To compare relative abundance of *MoSFA1* transcripts, average threshold cycle (Ct) was normalized to that of β-tubulin gene MGG_00604 as described by Livak & Schmittgen [28]. For the gene *MoSFA1*, primers GNO1-F3 and GNO1-R3, which amplify a 150-bp fragment from the *MoSFA1* coding region, were used. For the gene MGG_00604, the designed primers were Tub-F1 and Tub-R1, which specifically amplified a 156-bp DNA fragment from the β-tubulin coding region. All qPCR experiments were performed with three biological replicates.

### Determination of SNOs content

Total SNOs content in *M*. *oryzae* was determined using the Saville-Griess assay [29]. All tested strains were cultured in liquid CM for 3 days in the dark. Sodium nitroprusside (SNP), a NO donor, was added to cultures of each strain at a final concentration of 100 μM for an additional day at 2 days after incubation. Cultures were harvested and washed three times with sterile distilled water and ground in liquid nitrogen. The cell extracts were prepared in 50 mM Tris-HCl (pH 8.0) in the dark and incubated for 10 min in solution A (1% sulfanilamide in 0.5 M HCl) or solution B (solution A plus 0.2% HgCl_2_). The colored azo dye was formed after the incubated solutions reacting with an equal volume of solution C (0.02% (N-(1-naphthyl) ethylenediamine dihydrochloride in 0.5 M HCl) for 10 min. The absorbance was measured at 540 nm in a Thermo Varioskan Flash spectrophotometer. The SNOs content was determined according to the difference in absorbance between the reaction with solution B and that with solution A by comparing with those of a standard curve constructed using GSNO. The concentration of protein extract was determined by a Bradford assay method, with BSA as the standard. The resultant SNOs content was expressed as pmol per mg protein.

### Determination of antioxidant enzymes activities and GSH content

To determinate the activities of antioxidant enzymes and GSH content, cultures grown in liquid CM for 3 days were harvested and washed three times with sterile distilled water, then ground in liquid nitrogen. The cell extracts were prepared in 10 mM phosphate buffer (pH 7.4). The activities of superoxide dismutase (SOD, EC 1.15.1.1), catalase (CAT, EC 1.11.1.6), and peroxidase (POD, EC 1.11.1.7) and the content of reduced GSH were detected using commercial assay kits purchased from Nanjing Jiancheng bioengineering institute (Nanjing, China). The concentration of protein extract was determined by a Bradford assay method, with BSA as the standard. The absorbance were read in a Thermo Varioskan Flash spectrophotometer, and enzyme activity and GSH content were calculated according to according to the manufacturer's manual, and expressed as U per mg protein and μmol per mg protein, respectively.

## Results

### Identification of *MoSFA1*

The protein sequence of *S*. *cerevisiae* SFA1 (P32771) was used to search *GFD* homologue gene of *M*. *oryzae* from *Magnaporthe* comparative database (http://www.broadinstitute.org/annotation/genome/magnaporthe_comparative/MultiHome.html) with the BlastP program. A predicted gene MGG_06011 exhibited the highest similarity to yeast *SFA1*. MGG_06011 was annotated as 1333 bp nucleotide length with three exons, and putatively encoded a protein of 381 amino acids. The deduced protein sequence of MGG_06011 showed high similarity to the sequence of S-(hydroxymethyl)glutathione dehydrogenase previously characterized from yeast (67.5% identity with *S*. *cerevisiae* SFA1), human (65.5% identity with the *Homo sapiens* ADH5), and *Arabidopsis* (61.6% identity with *Arabidopsis thaliana* ADH2).

According to site-directed mutagenesis studies and crystal structure analysis of *H*. *sapiens ADH5*, several highly conserved residues are specifically coordinated to the active site zinc, the binding of substrates and the ligand of binary coenzyme complex, including Cys44, Thr46, Asp55, Glu57, His66, Glu67, Gln111, Arg114, Cys173 and Lys283 [30–34]. Alignment with the sequences from yeast (SFA1, P32771), *H*. *sapiens* (ADH5, P11766) and *A*. *thaliana* (ADH2, Q96533) revealed that the known active residues were strictly conserved in the protein of MGG_06011, except for Gln111 instead of Gly, but this exception was consistent with yeast and *Arabidopsis* (S1 Fig.). We therefore, termed MGG_06011 as *MoSFA1*, a homolog of *S*. *cerevisiae SFA1*.

### Heterologous expression of *MoSFA1*

**Fig 1:**
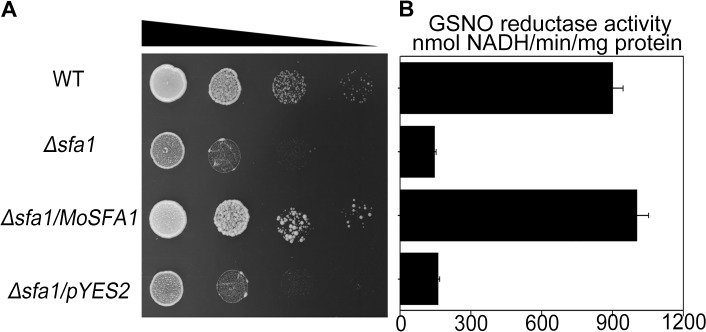
Complementation of the yeast *Δsfa1* mutant by *MoSFA1* and GSNO reductase activity in protein extracts. (A) Yeast strains, BY4741(WT), *Δsfa1*, *Δsfa1/*MoSFA1, and *Δsfa1/*pYES2 were spotted onto galactose-containing medium with 1.1 mM formaldehyde at 28°C for 2 days. (B) GSNO reductase activity (mean ±SD) was determined in the tested yeast strains. Error bars represent SD.

To obtain biochemical evidences of MoSFA1 protein in formaldehyde detoxification and GSNO reductase activity, we have constructed a plasmid pYES2-MoSFA1 containing the CDS of *MoSFA1* and consequently transformed the plasmid pYES2-MoSFA1 and empty vector pYES2 into yeast strain *Δsfa1*, respectively. Cells of yeast strain BY4741 and derivatives strains, *Δsfa1*, *Δsfa1*/MoSFA1 with pYES2-MoSFA1, and *Δsfa1*/pYES2 with pYES2, were serially diluted and plated on SD medium containing 1.1 mM formaldehyde for two days. Consistent with the expected, *Δsfa1*/pYES2-MoSFA1 regained the resistance to formaldehyde, compared with that of the wild type and of the *Δsfa1* strain (Fig. 1A).

Simultaneously, protein extracts from yeast were used to determine the GSNO reductase activity of *MoSFA1* recombinant protein. Our data showed that GSNO reductase activity of protein extracts from the *Δsfa1*/MoSFA1 and the wild type were 1002.1 and 899.2 nmol NADH consumed min^−1^ (mg protein)^−1^, respectively. However, GSNO reductase activities of protein extracts from both *Δsfa1* and *Δsfa1*/pYES2 were under 160 nmol NADH consumed min^−1^ (mg protein)^−1^ (Fig. 1B). Obviously, significant higher GSNO reductase activity of *Δsfa1*/MoSFA1 strain should be attributed to the *MoSFA1* introduction.

Taken together, our data indicated that *MoSFA1* gene functionally complemented the defect of *Δsfa1* strain, and confirmed that *MoSFA1* is the homolog of *SFA1* and the encoded protein has formaldehyde detoxification and GSNO reductase activity.

### Generation of *MoSFA1* deletion mutants

In order to investigate the function of *MoSFA1* in *M*. *oryzae*, the *MoSFA1* gene was disrupted. A *MoSFA1* gene replacement vector pKO-MoSFA1 was constructed. Conidia of Guy11 were used to genetic transformation by *A*. *tumefaciens*-mediated transformation, and transformants were screened twice in CM plates supplemented with 200 μg ml^−1^ hygromycin B. Homologous recombination of the *MoSFA1* replacement transformants was prescreened by PCR, then Southern blot data showed a single 3.1-kb band for Guy11 and another single 5.8-kb band for candidate transformants (G23 and G24) (S2A-B Fig.). The blotting pattern was consistent with expected gene replacement event at the *MoSFA1* gene locus. Thus, the *MoSFA1* deletion mutants, G23 and G24, were selected for further functional analysis in this study. We constructed a complementation strain G23R by reintroducing the *MoSFA1* genomic DNA sequence, including a 1.8-kb upstream sequence, *MoSFA1* gene open reading frame, and 0.4-kb downstream sequence, into the mutant G23. Relative transcript analysis confirmed the gene replacement event in G23 and G24, and that *MoSFA1* reintroducing in G23R was functional, with the evidences that expression of *MoSFA1* gene in G23R was same as that of Guy11 and *MoSFA1* was not transcribed in the gene deletion mutants (S2C Fig.).

### *MoSFA1* deletion mutants are lethal in formaldehyde-containing medium

**Fig 2:**
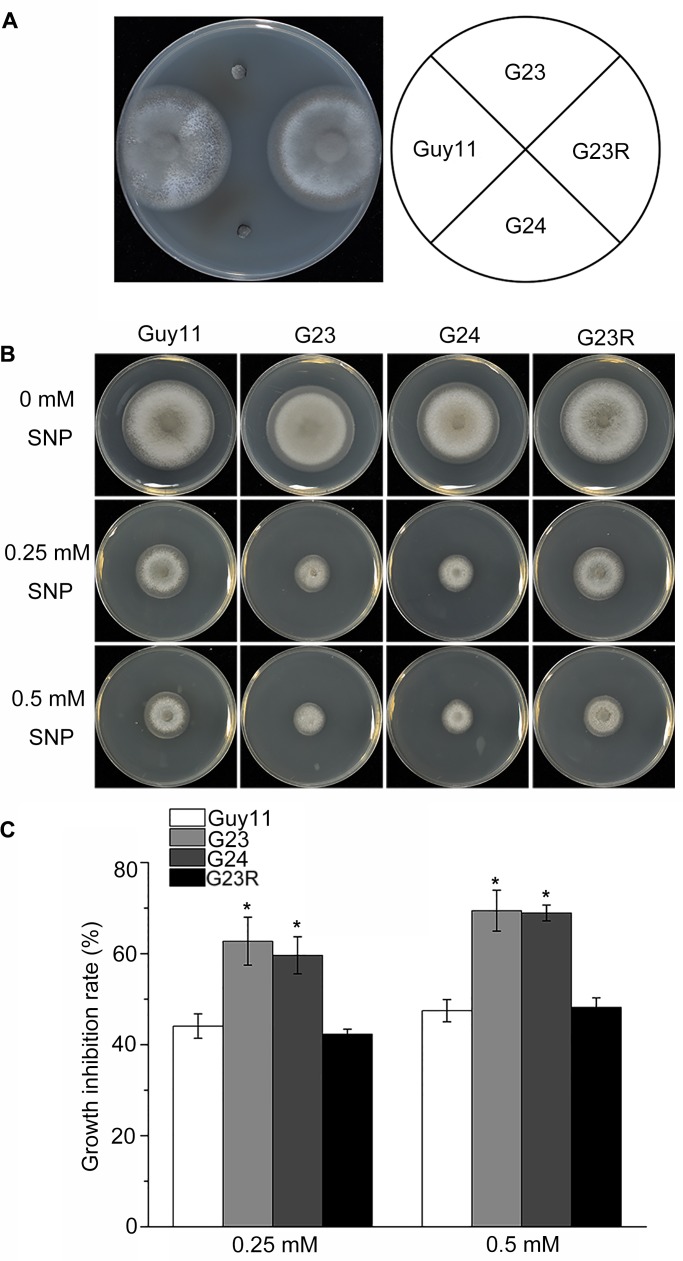
Effect of formaldehyde and SNP on vegetative growth of the tested *M*. *oryzae* strains. (A) *M*. *oryzae* strains were cultured on CM containing 0.3 mM formaldehyde at 28°C for 6 days. (B) Vegetative growth of Guy11, *MoSFA1* deletion and reintroduction mutants were grown on CM containing 0.25 or 0.5 mM SNP at 28°C for 6 days. (C) Colony diameters of the tested strains were measured and Growth inhibition rate were evaluated. The experiments were performed in triplicate. ANOVA analysis was performed after growth inhibition rate were arcsine transformed. However, the original percentages were used for presentations. Error bars represent SD of the original percentages. Asterisks in each data column indicate significant differences at p = 0.05.

To establish whether *MoSFA1* is also involved in formaldehyde metabolism in *M*. *oryzae*, formaldehyde sensitivity of *MoSFA1* deletion mutants was tested. Growth of *MoSFA1* deletion mutants were completely inhibited on CM containing 0.3 mM formaldehyde, compared with that of Guy11 and G23R (Fig. 2A). Loss of *MoSFA1* resulted in lethal phenotype in the presence of formaldehyde, indicating that *MoSFA1* in *M*. *oryzae* is necessary for formaldehyde detoxification.

### *MoSFA1* deletion mutants shows sensitivity to exogenous NO

The *MoSFA1* deletion mutants were biochemically evaluated for the resistance to NO stress in comparison with the wild type. All tested strains were exposed to different concentrations of SNP for 6 days. Growth of all tested strains were reduced in the CM plates with SNP treatment. In particular, *MoSFA1* deletion mutants showed more severe growth inhibition (Fig. 2B). The rates of growth inhibition was increased by 17% and 22% in the mutants as compared with Guy11 after exposure to 0.25 and 0.5 mM SNP, respectively (Fig. 2C). Defect of resistance to SNP in *MoSFA1* deletion mutants was recovered by reintroducing the gene (Fig. 2B and 2C). These results further confirmed that *MoSFA1* is involved in resistance to NO stress.

### *MoSFA1* deletion results in significant increase of SNOs content in cells

**Fig 3:**
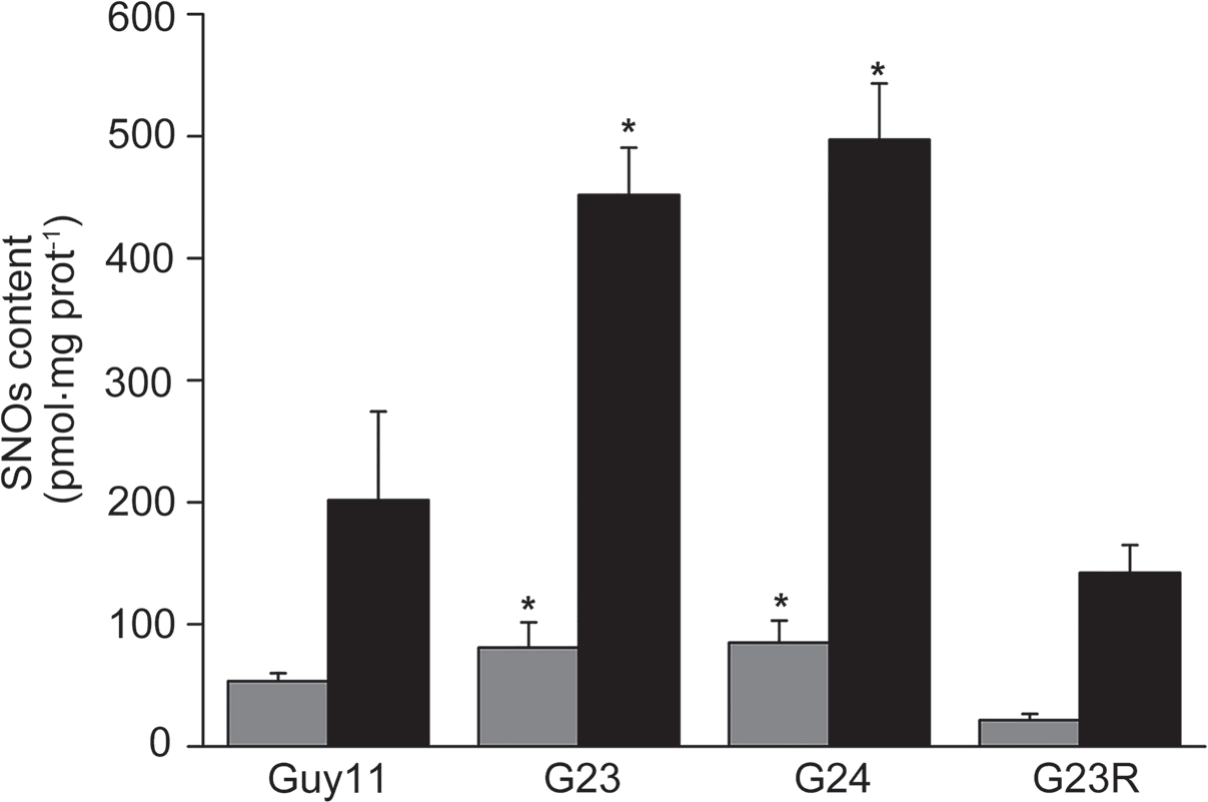
The levels of SNOs in the tested *M*. *oryzae* strains. Total intracellular SNOs content in mycelia cultured in liquid CM in the dark at 28°C for 3 days without (gray bar) or with 100 μM SNP treatment (black bar). The values obtained were compared to a standard curve constructed using GSNO. The results were normalized to the protein content by a Bradford assay. The experiments were performed in triplicate. Error bars represent SD. Asterisks in each data column indicate significant differences at p = 0.05.

In yeast, loss of *SFA1* results in increase of cellular SNOs [16, 17]. To compare SNOs content between *MoSFA1* deletion mutants and Guy11, protein extracts from the tested strains without or with SNP treatment were used to determine the levels of SNOs. As shown in Fig. 3, the basal levels of SNOs in *MoSFA1* deletion mutants reached more than 80 pmol mg^−1^ protein and were about 1.5 times higher than that of Guy11 [53.4 pmol mg^−^1 protein] (Fig. 3). With SNP treatment, cellular SNOs contents in all tested strains were significantly increased, against that without SNP treatment. Notably, the SNOs contents in *MoSFA1* deletion mutants were at least two fold higher as compared with that of Guy11 [201.8 pmol mg^−^1 protein]. More interestingly, SNOs content in *MoSFA1* reintroduced mutant was reduced regardless of with or without SNP treatment compared with that of gene deletion mutants, and was even lower than that of the wild type. Taken together, loss of *MoSFA1* in *M*. *oryzae* led to increase of SNOs content, which implied that additional protein S-nitrosylation had occurred to subsequently lead to change in physiological and biological functions in *MoSFA1* deletion mutants.

### *MoSFA1* is required for normal vegetative growth and conidiation

The two gene deletion mutants were analyzed in order to detect any possible alteration in normal vegetative growth and conidiation. Our data showed that *MoSFA1* deletion mutants grew slowly with whiter colony and looser aerial hyphae, compared with Guy11 (S3A-B Fig.). Moreover, reduction of black pigments in the mutants was more obvious in liquid cultures. Cultures of Guy11 produced more abundant black pigments in the dark after 5 days, compared with the *MoSFA1* deletion mutants (S3C Fig.).

**Fig 4:**
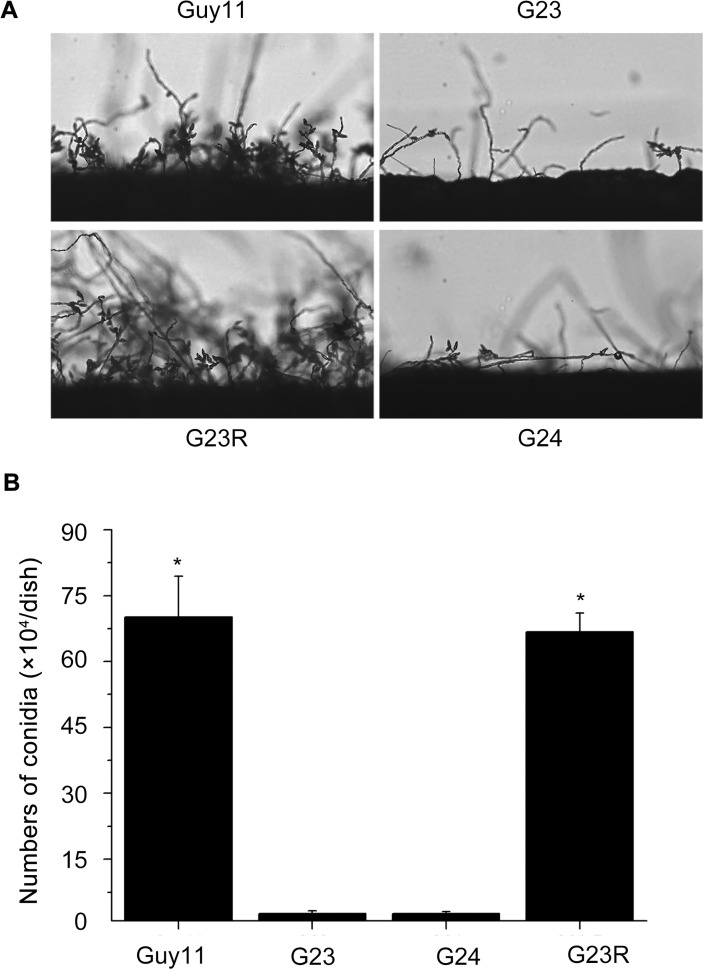
Comparison of the tested *M*. *oryzae* strains in conidiation. (A) Development of conidia on conidiophores on CM. Conidia and conidiophores were observed under light microscopy. (B) Statistical analysis of the number of conidia in each 9-cm-diameter dish. The experiments were performed in triplicate. Error bars represent SD. Asterisks in each data column indicate significant differences at p = 0.05.

By counting the conidia of the tested strains grown on 9-day-old CM plates with 12 hour light and dark alternation under fluorescent light, conidial production was significantly reduced in the *MoSFA1* deletion mutants compared with that of the wild type. As shown in Fig. 4A and 4B, conidia produced in *MoSFA1* deletion mutants was only ~2% as compared with the wild type. All defects described above were recovered though reintroducing *MoSFA1* gene into the mutant G23. The results suggested that *MoSFA1* is involved in normal vegetative growth and conidiation of *M*. *oryzae*.

### *MoSFA1* deletion attenuates the virulence of *M*. *oryzae*

**Fig 5:**
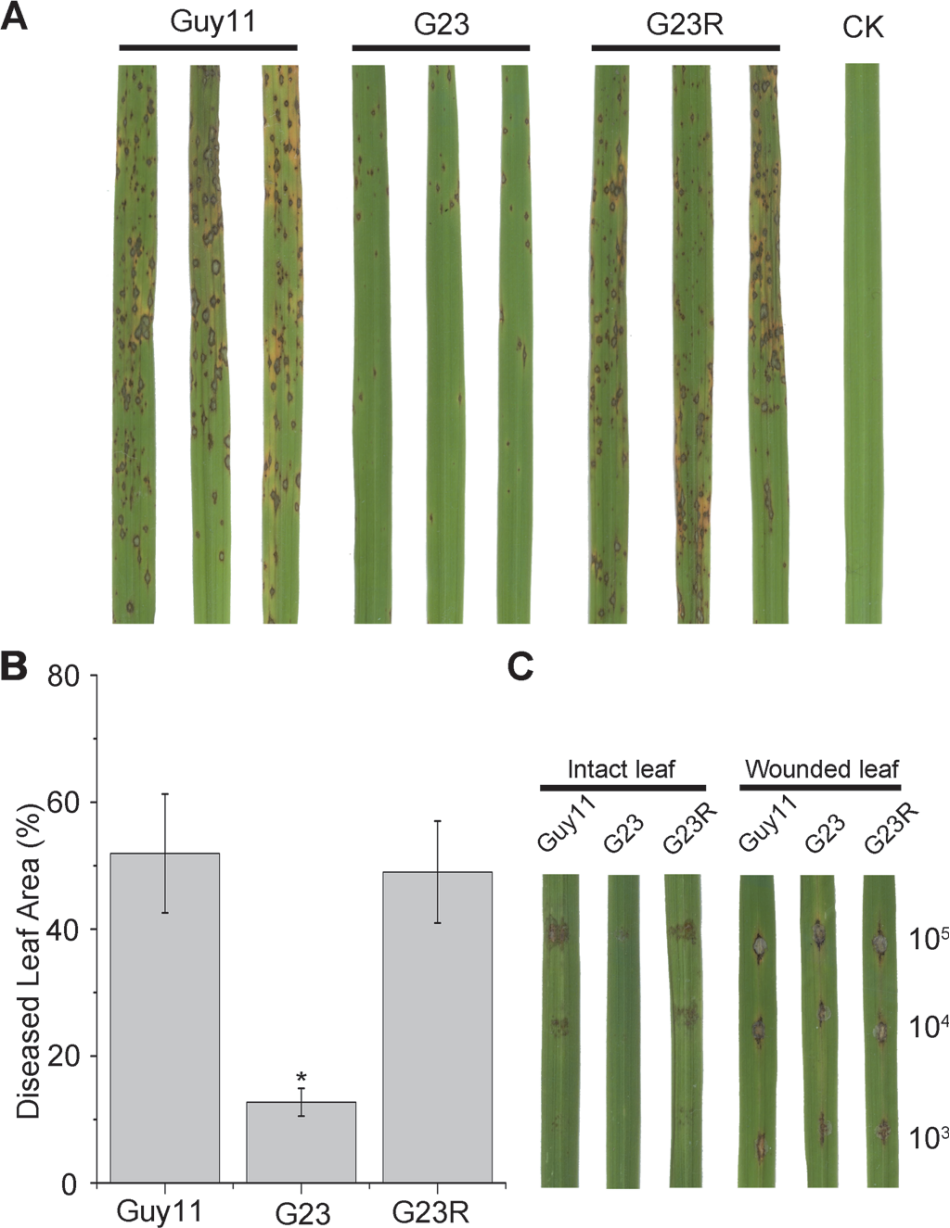
Pathogenicity assays on rice leaves. (A) Spray inoculation with 4-week-old rice seedlings. Rice leaves (*O*. *sativa* cv. CO-39) were inoculated with conidia at a concentration of 5×10^4^ conidia ml^−1^. Representative leaves were photographed 6 days post-inoculation. The experiments were repeated at least three times with triple replications that yielded similar results. (B) Disease severity of each strain was assessed from the percentage of diseased leaf area as described by Fang and Dean (2000). ANOVA analysis was performed after percentages were arcsine transformed. However, the original percentages were used for presentations. Error bars represent SD of the original percentages. Asterisks in each data column indicate significant differences at p = 0.05. (C) Drop inoculation with 20 μl serial dilutions of conidia suspension on intact and wounded rice leaf segments. Representative leaves were photographed 6 days post-inoculation.

To determine the virulence of the *MoSFA1* deletion mutant, 4-week-old susceptible rice seedlings were used for pathogenicity assays. Rice leaves were sprayed with conidial suspensions at the concentration of 5×10^4^ conidia per ml to develop blast lesions. Six days after inoculation, symptoms on rice leaves had fully emerged, and the leaves inoculated with *MoSFA1* deletion mutant displayed fewer lesions compared with those inoculated Guy11 (Fig. 5A). The estimated disease lesion area caused by the *MoSFA1* mutants occupied about 40% less of the leaf surface than the lesion area caused by the wild-type strain Guy11 (Fig. 5A and 5B). The attenuated virulence of the *MoSFA1* mutants could be complemented and fully recovered by reintroducing the *MoSFA1* gene. The results suggested that *MoSFA1* is required for full virulence of *M*. *oryzae*.

In order to determine whether *MoSFA1* is involved in the virulence in biotrophic or necrotrophic phase, a wounded inoculation in rice leaf was performed. The results showed no significant difference in virulence among the tested strains (Fig. 5C). Taken together, our finding implies that *MoSFA1* significantly contributes to virulence in penetration or biotrophic phases, not in necrotrophic phase.

### *MoSFA1* deletion results in decreased appressorial turgor pressure and retarded growth of infectious hyphae

**Fig 6:**
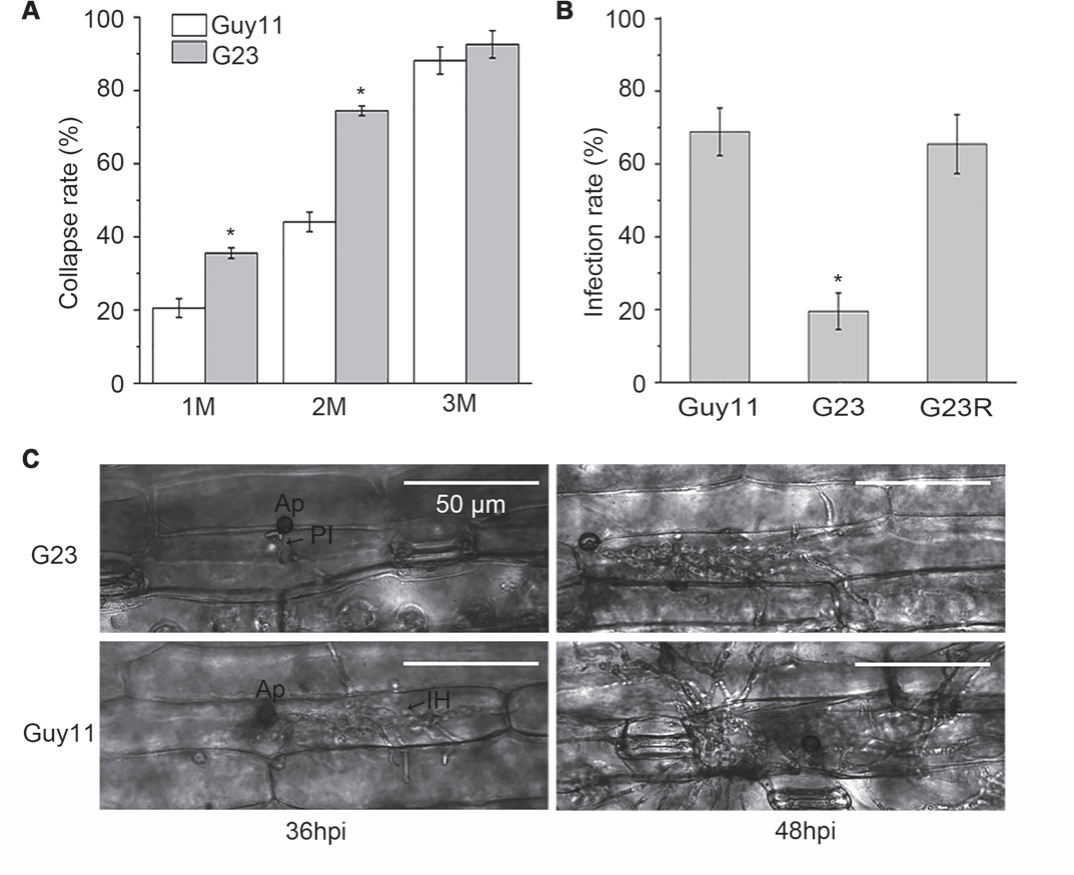
Analysis of infection-related morphogenesis of *MoSFA1* deletion mutant. (A) Cytorrhysis assay using glycerol was performed to compare the appressorial turgor pressure of *MoSFA1* mutant and wild-type strain. Different glycerol solutions were given to appressoria at 48 hpi. Appressorial cytorrhysis was counted under an optical microscope. The rate of cytorrhysis was the average of three replications. (B) Infection rate of *M*. *oryzae* strains in barley leaf cells at 36 hpi. (C) Growth of infectious hyphae of *MoSFA1* deletion mutant was retarded in barley leaf cells. Penetration and infectious hyphae were examined under optical microscopy. Ap, appressorium; PI, primary infectious hyphae; IH, secondary infectious hyphae. Significant difference analysis was performed after percentages were arcsine transformed. The original percentages were used for presentations. Error bars represent SD. Asterisks in each data column indicate significant differences at p = 0.05.

To further explore why *MoSFA1* deletion attenuates the virulence of *M*. *oryzae*, infection-related morphogenesis of the *MoSFA1* deletion mutant was investigated on both artificial hydrophobic interface and barley leaf. Conidia of *MoSFA1* deletion mutants normally germinated and differentiated into melanized appressoria, and the rates of conidial germination and appressorial formation were not affected evidently, compared with the wild-type strain (S4 Fig.). However, appressorial collapse rate of the *MoSFA1* deletion mutants were significantly higher in 1 M and 2M glycerol solution than of the wild type (Fig. 6A). The fact suggested that loss of *MoSFA1* results in reduction of appressorial turgor pressure.

Furthermore, infection rates of *MoSFA1* deletion mutants in barley leaf were observed and statistically analyzed. And *MoSFA1* deletion mutants significantly caused fewer infection events compared to the wild type. More than 68% of the appressoria of the wild type strain formed primary infectious hyphae at 36 hpi, but the infection rate was only about 20% in *MoSFA1* deletion mutant (Fig. 6B).

Meanwhile, we also found that infectious hyphae growth of *MoSFA1* deletion mutants were severe delayed in barley cells (Fig. 6C). As shown in Fig. 6C, growth and expansion of infectious hyphae from the point of infection were significantly attenuated at 36 hpi and 48 hpi compared to the wild type strain.

Taken together, our data suggested that *MoSFA1* contributes significantly to appressoria maturation and infectious hyphae development in host cells.

### Accumulation of H_2_O_2_ in cells infected by *MoSFA1* deletion mutants

**Fig 7:**
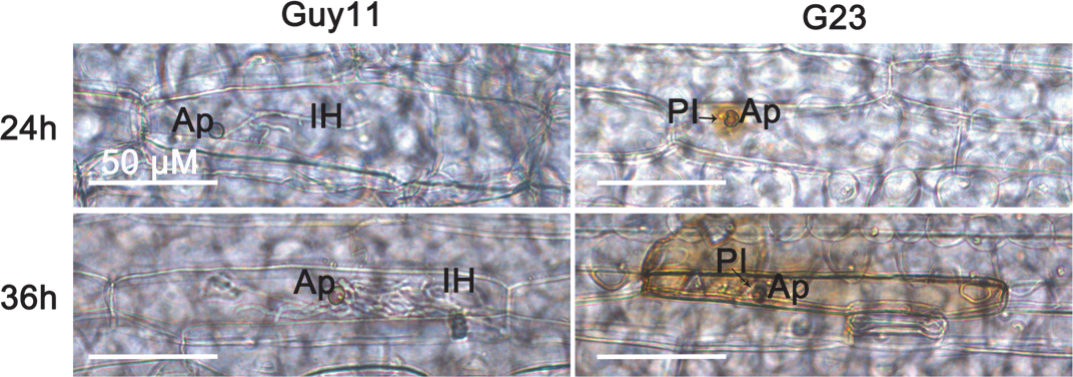
Detection of H_2_O_2_ accumulation at infected barley cells. Detached barley leaf were inoculated with the tested strains, stained with DAB at 24 and 36 hpi, and observed under the light microscope. The cells infected by *MoSFA1* deletion mutant were strongly stained with DAB, indicating high H_2_O_2_ accumulation at the penetration site. Bar is 50 μm. Ap, appressorium; PI, primary infectious hyphae; IH, secondary infectious hyphae.

Defense responses of hosts induced by pathogen often associated with the rapid production of ROS, which form a barrier to penetration and inhibit the growth of pathogen directly. Thus, the host ROS burst which responded against the wild type and the mutants were compared. As shown in Fig. 7, the accumulation of H_2_O_2_ at infected barley cells by *MoSFA1* deletion mutants were stronger when stained with DAB-staining, compared with the infections of Guy11 and G23R (Fig. 7). These data suggest that loss of *MoSFA1* gene in *M*. *oryzae* resulted in accumulation of H_2_O_2_ in infected host cells, which may contribute retarded growth of infectious hyphae in host cells.

### *MoSFA1* deletion mutants shows hypersensitivity to oxidative stress

**Fig 8:**
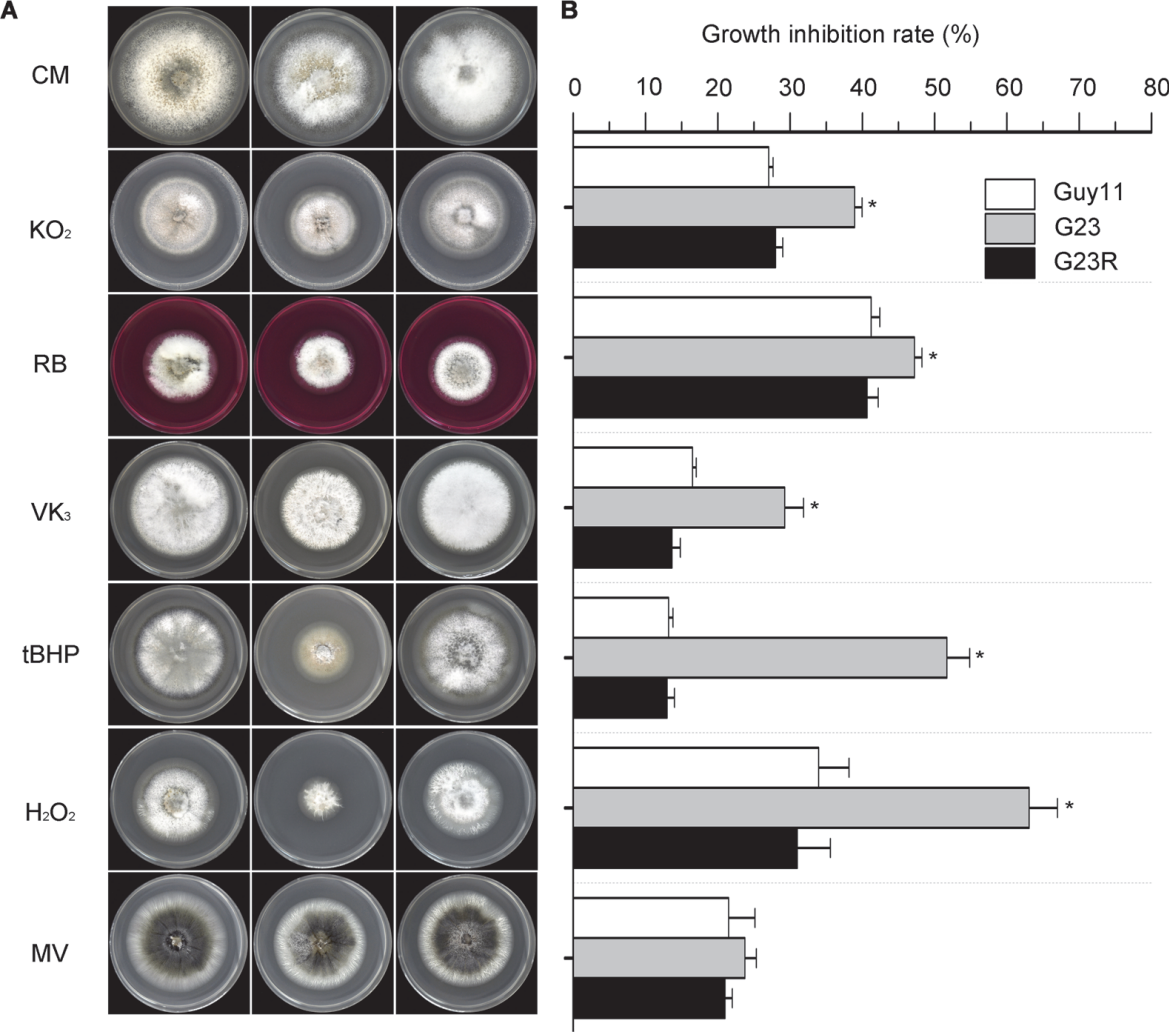
Effect of different oxidants on vegetative growth of the tested *M*. *oryza*e strains. (A) Vegetative growth of Guy11, *MoSFA1* deletion and reintroduction mutants were grown on CM containing menadione (VK_3_, 100 μM), potassium superoxide (KO_2_, 50 mM), rose bengal (RB, 100 μM), tert-butyl-hydroxyperoxide (tBHP, 0.001%,v/v), and H_2_O_2_ (0.04%, v/v) and Methyl viologen (MV, 0.25mg/ml) at 28°C for 6 days. (B) Growth inhibition rate of tested strains exposed to different oxidants. The experiments were performed in triplicate. ANOVA analysis was performed after growth inhibition rate were arcsine transformed. However, the original percentages were used for presentations. Error bars represent SD. Aasterisks in each data column indicate significant differences at p = 0.05.

To test a possibility that ROS play a role in the delayed growth of *MoSFA1* mutant in host cells, *M*. *oryzae* strains were exposed to different oxidants. Growth of *MoSFA1* deletion mutants were inhibited on CM containing the tested oxidants, compared with that of Guy11. Growth of *MoSFA1* deletion mutant was inhibited by 29.3% to 63.1% in the presence of 100 μM menadione, 50 mM potassium superoxide, 100 μM rose Bengal, 0.001% (v/v) tert-butyl-hydroxyperoxide, and 0.04% (v/v) H_2_O_2_, and the growth inhibition rates were significantly more greater than of Guy11 (Fig. 8A and 8B). Methyl viologen (0.25mg/ml) also inhibited growth of the tested strains, but no significant difference in growth inhibition rate was found statistically. The defects to all tested oxidants were recovered by reintroducing *MoSFA1* in the mutant G23. Our data suggested that loss of *MoSFA1* gene in *M*. *oryzae* causes reduced resistance to oxidative stress.

### Loss of *MoSFA1* reduces the activities of antioxidant enzymes and GSH content in cells

**Fig 9:**
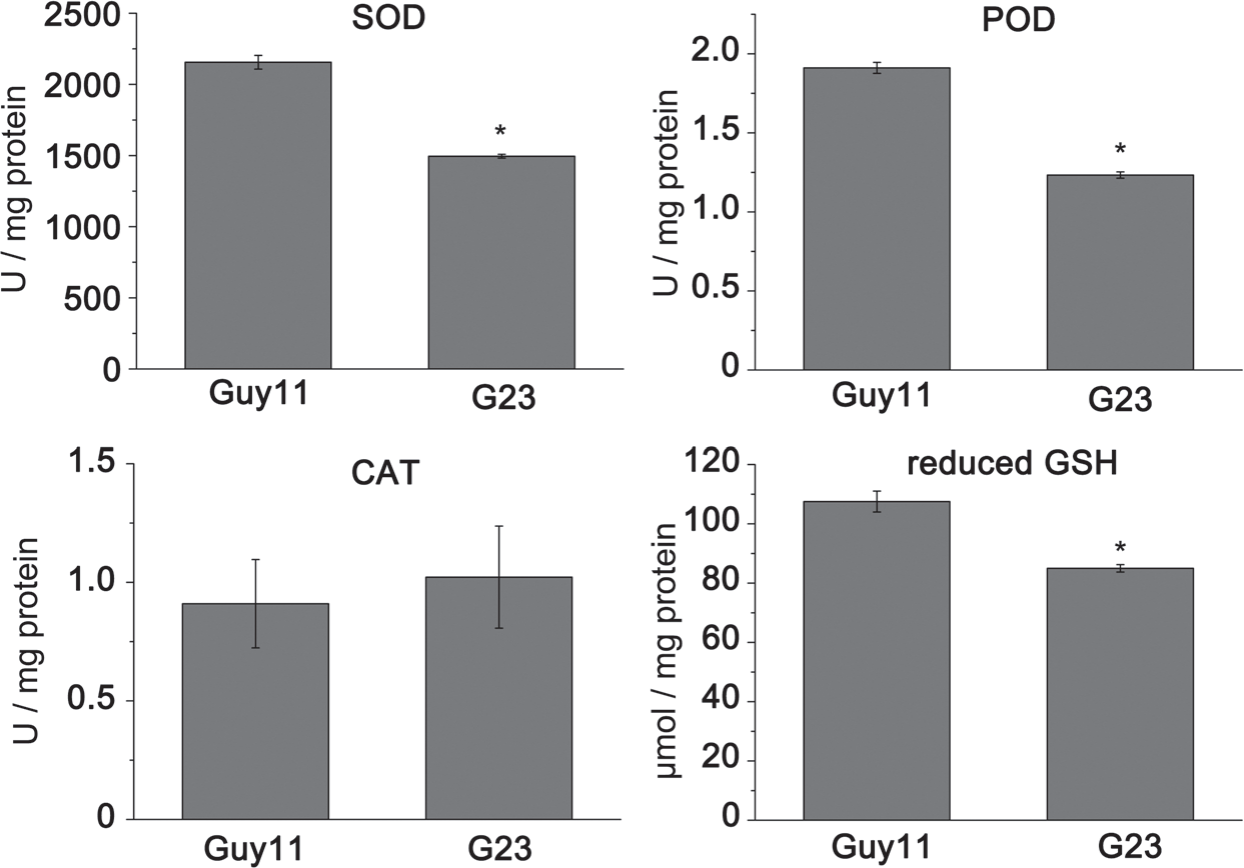
The activity of antioxidant enzymes and the content of GSH in tested *M*. *oryzae* strains. Activities of superoxide dismutase (SOD), catalase (CAT), and peroxidase (POD) and the content of reduced GSH in the tested strains were determined using assay kits purchased from Nanjing Jiancheng Bioengineering Institute (Nanjing, China). The experiments were performed in triplicate. Error bars represent SD. Asterisks in each data column indicate significant differences at p = 0.05.

Considering the hypersensitivity of *MoSFA1* deletion mutants to oxidants, *MoSFA1* were presumed to involve in redox homeostasis in cells. Superoxide dismutases, catalases, and peroxidases provide the cells with highly efficient machinery for detoxifying ROS [35]. GSH acts as a radical scavenger and an electron donor, and the concentration of GSH in cells is important to keep redox homeostasis [36]. Our data showed that the activities of SOD and POD in *MoSFA1* deletion mutant had significantly lower levels than those of the wild type, but CAT activity was not different from that of the wild type. In addition, the concentration of reduced GSH in *MoSFA1* deletion mutant was significantly diminished in cells (Fig. 9).

## Discussion

NO produced by the plant plays crucial roles in resistance to pathogen attack, and NO bursts in rice cells has been discovered after treatment with compatible or incompatible blast fungus elicitor, which is required for induction of cell death and defense gene activation [5]. However little is documented about the defense mechanisms that *M*. *oryzae* is armed to protect against NO-relative molecules. This work shows that *MoSFA1*, a homolog of S-(hydroxymethyl)glutathione dehydrogenase gene *SFA1* from yeast which specifically catalyze the reduction of GSNO to involve in NO metabolism, is required for conidiation and full virulence.

In present study, *MoSFA1* deletion mutants exhibited reduced infection and growth of infectious hyphae (Fig. 6B and 6C). Moreover, the strong accumulation of H_2_O_2_ in infected cells were observed by inoculation of *MoSFA1* deletion mutant (Fig. 7). However, the induction levels of PR genes in challenged rice tissue by *MoSFA1* deletion mutant were not different from those in challenged rice tissue by the wild type (S5 Fig.). It suggests that *MoSFA1* in *M*. *oryzae* is involved in compromising the host defence-related oxidative burst in compatible interaction. Enzyme activity assay showed that the activities of SOD and POD in *MoSFA1* deletion mutant were significantly inhibited, and the concentration of reduced GSH were significantly less than that in wild type (Fig. 9). Thus, the defect in virulence of *MoSFA1* deletion mutants is due to the impairment of the ability to maintain redox homeostasis. Firstly, GSH is important for the maintenance of the redox homeostasis [37], and production of GSNO need to consume GSH. Loss of *MoSFA1* not only repeals the reduction of GSNO to GSSG but also causes the additional GSH consumption attributing NO administration, which subsequently disturbs with the redox balance of GSH in cells. Recently, GSH-recycling in GSH-dependent antioxidant system of *M*. *oryzae* is discovered to be critical in biotrophic colonization of host cells [38]. Loss of *GTR1* encoding glutathione reductase significantly retarded growth of infectious hyphae in rice cells and reduced the full virulence. Secondly, GSNO acts as the main NO reservoir in cells and can directly release NO. GSNO accumulation leads to cellular NO stress, and NO can reduce the enzymatic activity to oxidative detoxification [13, 39–42]. Specifically, NO can reversibly inhibit the cytochrome c oxidase enzyme to block respiratory chain and to markedly increase the production rate of ROS [41]. NO administration also reduces the activities of superoxide dismutase and catalase, increases the level of intracellular ROS and secondarily leads to growth retardation in *Penicillium expansum* [13]. Additionally, NO can rapidly react with oxygen species, such as superoxide [43], to form the peroxynitrite (ONOO^-^), which is a powerful oxidant and reacts with a wide array of molecules in cells, including DNA, lipids and proteins, leading to cellular damage and cytotoxity [44].

Additionally, GNSO can transfer NO group to other cellular thiols-containing proteins to form S-nitrosothiols, which is one of the most important functional forms of NO-dependent posttranslational modification. Several proteome-wide analyses showed that a large spectrum of proteins are S-nitrosylated, including enzymes, structural proteins, and transcription factors [45–48]. Functional significance of protein S-nitrosylation in plants have been discovered, such as GAPDH (glyceraldehyde-3-phosphate dehydrogenase) [49], NPR1 (nonexpressor of pathogenesis-related gene1), the transcription factor TGA1 (a DNA-binding protein TGA1-like protein) [50, 51], and NADPH oxidase [52], and cytosolic ascorbate peroxidase [41]. In *Arabidopsis*, S-(hydroxymethyl)glutathione dehydrogenase gene regulates global levels of SNOs, and loss of this gene leads to the increased basal levels of SNOs and causes pleiotropic effects in abiotic or biotic resistances and development, and so on. [53–55]. The facts explain that SNOs are important mediators in the above-mentioned biological process in plants. This explanation also is supported by the findings in S-nitrosylated proteins identified in *noe1* rice, which accumulated more SNOs in comparison with wild-type plants and emerges a light-induced leaf cell death. Total 121 nitrosylated proteins were identified from wild-type and *noe1* plants, which are involved in different processes, including general metabolism, environmental adaptation, genetic information processing, and redox regulation. Among those, twenty-one and forty-eight proteins were identified only from wild type and *noe1* plants, respectively [48]. Similarly, we found that loss of *MoSFA1* in *M*. *oryzae* leaded to the increased basal level of SNOs, which was ~1.5 times higher than that of the wild type. Moreover, the level of SNOs induced by NO stress was higher in *MoSFA1* deletion mutants compared with wild-type strain (Fig. 3). As mentioned above, the difference of S-nitrosylated proteins between *MoSFA1* mutants and the wild type strain may result in the changes of gene expression and enzymes activity, which respond to the tested defect of *MoSFA1* deletion mutants. It implies that *MoSFA1*-mediated SNOs homeostasis is important to regulate normal biological function in *M*. *oryzae*.

Simultaneously, endogenous NO in fungi is discovered, and found to be involved in conidiation [56, 57], spore germination [58], and formation of infection structure [59]. Recently, Samalova et al. have demonstrated that endogenous NO in *M*. *oryzae* is crucial to the development and initiation of infection [60]. According to the findings, endogenous NO was accumulated and consumed during these biological processes. Thus, NO metabolism should be equally important to regulate specific function of NO in fungi.

Collectively, loss of *MoSFA1* increases the level of SNOs, which indirectly leads to NO stress. It suggests that *MoSFA1*-mediated NO metabolism is important in redox homeostasis and protein S-nitrosylation in response to development and host infection of *M*. *oryzae*. However, the mechanisms by which proteins is S-nitrosylated and how NO inhibits the antioxidant system in *M*. *oryzae* are still needed to understand in details, which may help to reveal the role of NO function in this fungus.

## Supporting Information

S1 Fig Amino acid sequence alignment of S-(hydroxymethyl)glutathione dehydrogenase. The amino acid sequence alignment of S-(hydroxymethyl)glutathione dehydrogenase from *M*. *oryzae* MoSFA1 (this study), *S*. *cerevisiae* (P32771), *H*. *sapiens* (P11766), and *A*. *thaliana* (Q96533) was performed. Identical amino acids in all sequences are shaded black, conservative replacements are shaded gray. The conserved residues are marked with ▲.
(TIF)

S2 Fig Generation of the *MoSFA1* deletion mutants and reintroduction mutant. (A) The 2.1-kb fragment including *MoSFA1* coding region was replaced with the *hph* cassette by homologous recombination. A 1.5-kb fragment from the 3’-end of *MoSFA1* gene was amplified as the probe for Southern blotting. Scale bar = 1 kb. (B) DNA gel blot analysis of genomic DNA from Guy11 and 2 transformants (G23 and G24) digested with *Bst*XI using the digoxigenin-labeled probes as shown in A. A single 3.1-kb band for Guy11 and another single 5.8-kb band for gene replacement. (C) Transcript levels (mean ±SD) of *MoSFA1* in Guy11, *MoSFA1* deletion mutants and the reintroduction mutant by quantitative PCR.
(TIF)

S3 Fig Vegetative growth of the tested *M*. *oryzae* strains in CM medium. (A) Colonies of the strains cultured on CM plates for 9 days at 28°C with 12 h light and dark alternation. (B) Colony diameters of the tested strains were measured and then statistically analyzed. Error bars represent SD. Asterisks in each data column indicate significant differences at p = 0.05. (C) Ten thousand conidia were cultured in liquid CM with shaking at 150 rpm at 28°C for 5 days and then photographed.
(TIF)

S4 Fig The rates of spore germination and appressoria formation in the tested *M*. *oryzae* strains. The rates of germination and appressoria formation were evaluated at 28°C after 24 hpi, and >200 conidia of each strain were observed for each strain. The experiments were replicated three times. Blank bar for germination rate. Grey bar for appressoria formation rate.
(TIF)

S5 Fig The expression of rice pathogenesis-related (PR) genes after chanllege inoculation with *M*. *oryzae* strains. Four-week-old seedlings of rice (*O*. *sativa* cv CO-39) were inoculated with suspensions of *M*. *oryzae* conidia prepared in 0.25% gelatin at a concentration of 5×10^4^ conidia ml^−1^ using an artist's airbrush with high-pressure air. Rice leaves were sampled at 24, 48, and 72 hpi. The relative expression levels of *PR1a*, *PR4*, *PR5*, and *PR10a* in the infected rice was compared using quantitative RT-PCR. Normalization of average threshold cycle (Ct) was performed with that of *O*. *sativa* elongation factor 1α gene. Primers were synthesized according to Hao et al. (Plant Physiol Biochem. 2012; 60:150–156).
(TIF)

S1 Table Primers used in this study. (PDF)
