# Supplementary figures and images for: An S-(Hydroxymethyl)Glutathione Dehydrogenase Is Involved in Conidiation and Full Virulence in the Rice Blast Fungus Magnaporthe oryzae
Source: PLoS One. 2015 Mar 20;10(3):e0120627. doi: 10.1371/journal.pone.0120627 (PMC4368689; doi:10.1371/journal.pone.0120627)

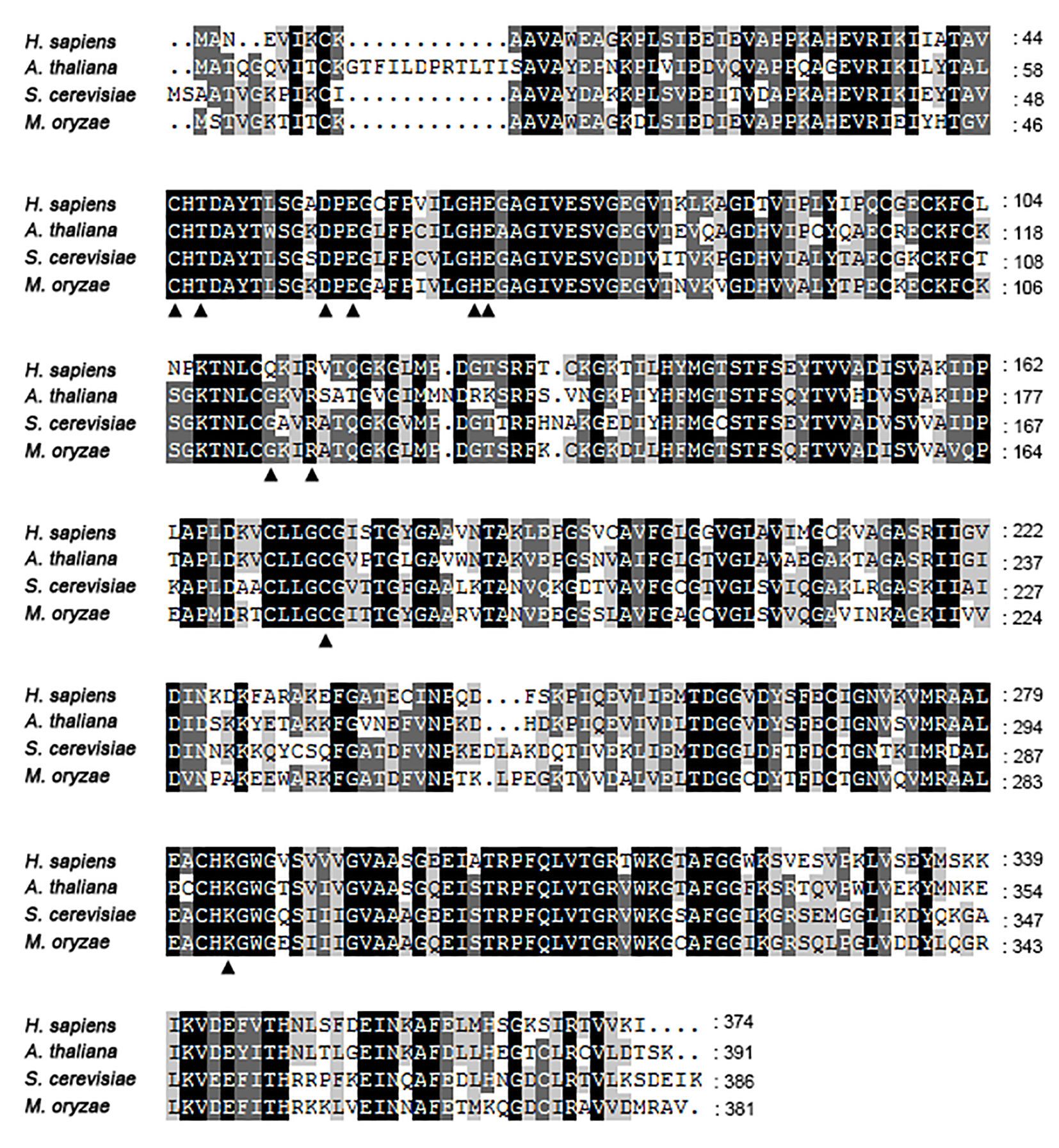

Supplement: S1 Fig — The amino acid sequence alignment of S-(hydroxymethyl)glutathione dehydrogenase from M. oryzae MoSFA1 (this study), S. cerevisiae (P32771), H. sapiens (P11766), and A. thaliana (Q96533) was performed. Identical amino acids in all sequences are shaded black, conservative replacements are shaded gray. The conserved residues are marked with ▲. (TIF) [file pone.0120627.s001.tif]

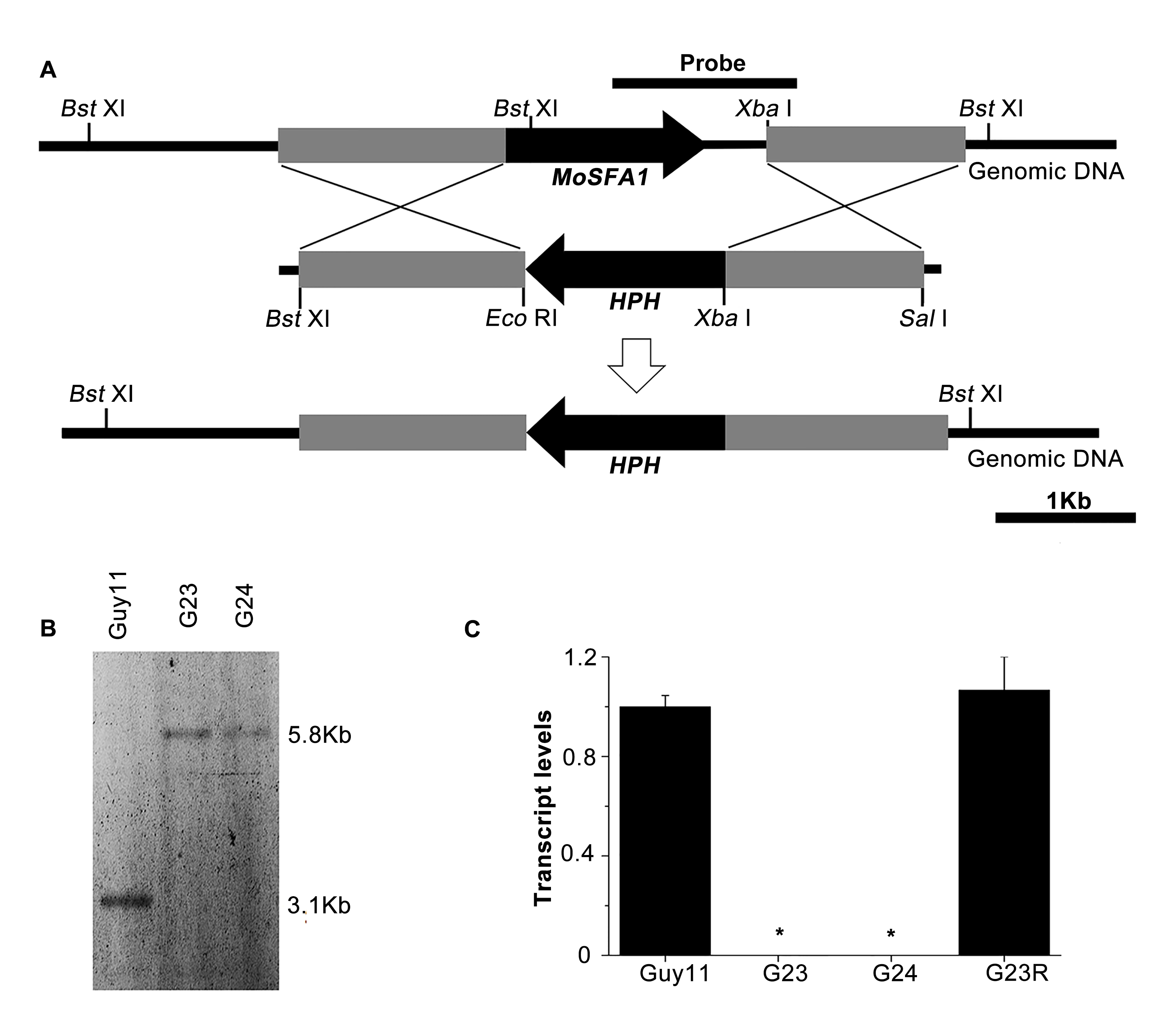

Supplement: S2 Fig — (A) The 2.1-kb fragment including MoSFA1 coding region was replaced with the hph cassette by homologous recombination. A 1.5-kb fragment from the 3’-end of MoSFA1 gene was amplified as the probe for Southern blotting. Scale bar = 1 kb. (B) DNA gel blot analysis of genomic DNA from Guy11 and 2 transformants (G23 and G24) digested with BstXI using the digoxigenin-labeled probes as shown in A. A single 3.1-kb band for Guy11 and another single 5.8-kb band for gene replacement. (C) Transcript levels (mean ±SD) of MoSFA1 in Guy11, MoSFA1 deletion mutants and the reintroduction mutant by quantitative PCR. (TIF) [file pone.0120627.s002.tif]

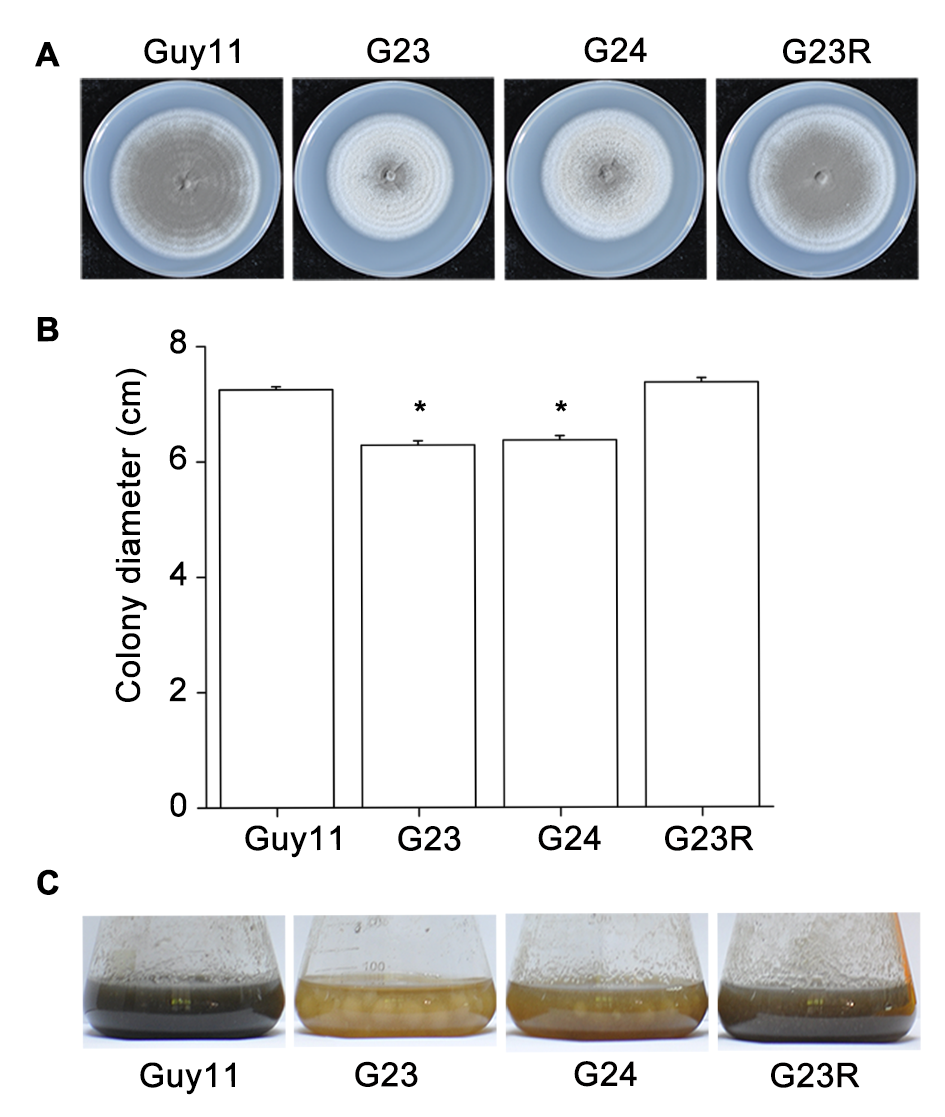

Supplement: S3 Fig — (A) Colonies of the strains cultured on CM plates for 9 days at 28°C with 12 h light and dark alternation. (B) Colony diameters of the tested strains were measured and then statistically analyzed. Error bars represent SD. Asterisks in each data column indicate significant differences at p = 0.05. (C) Ten thousand conidia were cultured in liquid CM with shaking at 150 rpm at 28°C for 5 days and then photographed. (TIF) [file pone.0120627.s003.tif]

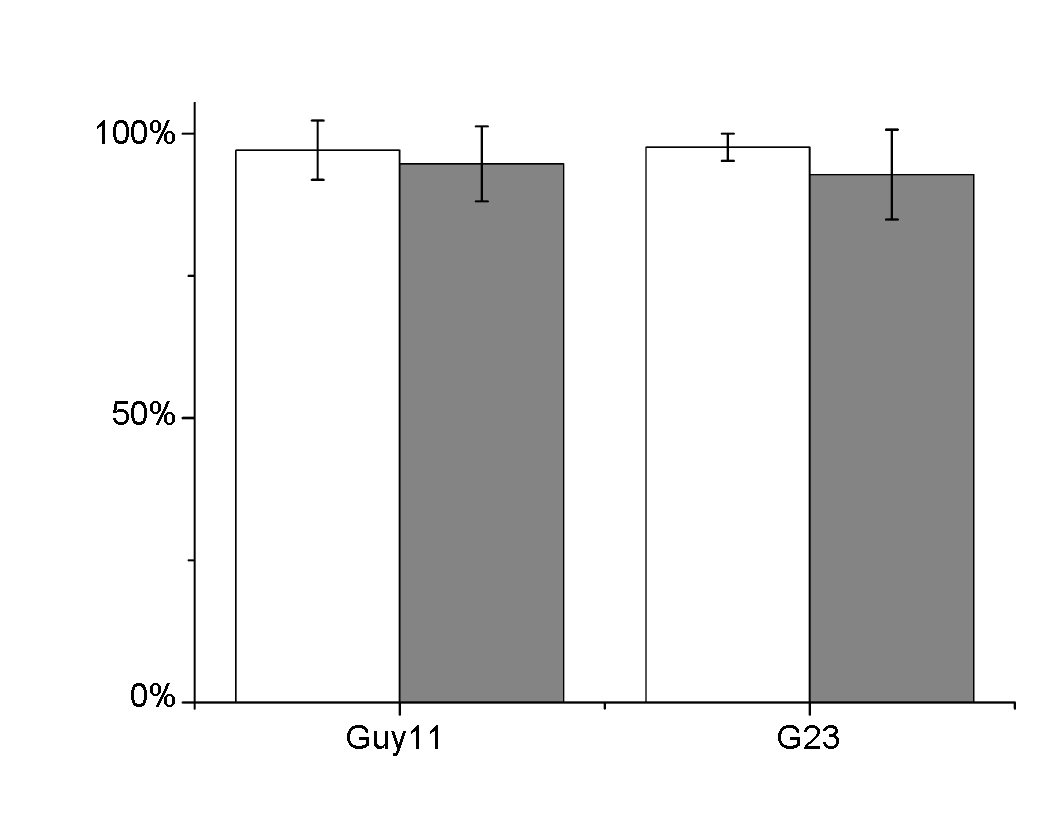

Supplement: S4 Fig — The rates of germination and appressoria formation were evaluated at 28°C after 24 hpi, and >200 conidia of each strain were observed for each strain. The experiments were replicated three times. Blank bar for germination rate. Grey bar for appressoria formation rate. (TIF) [file pone.0120627.s004.tif]

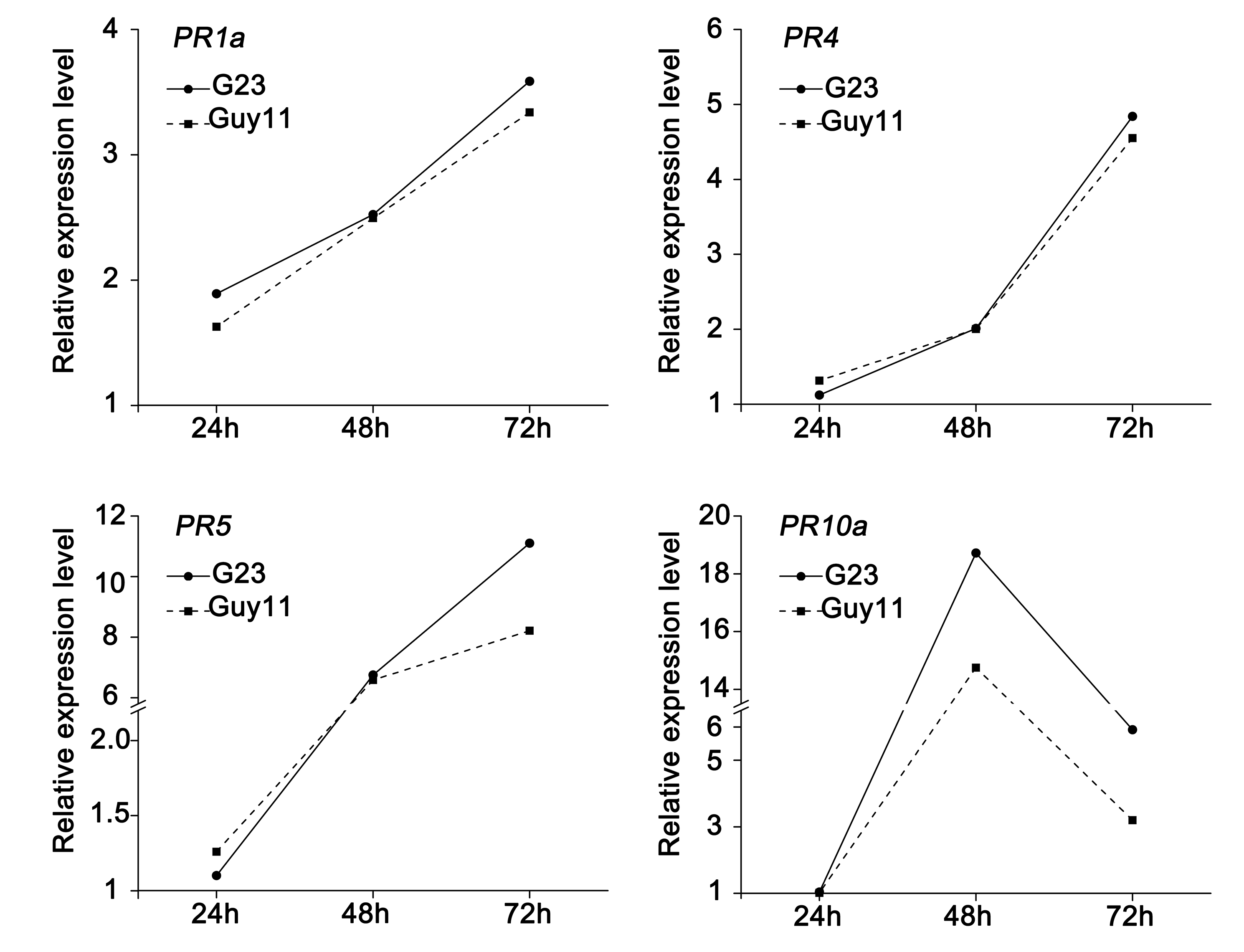

Supplement: S5 Fig — Four-week-old seedlings of rice (O. sativa cv CO-39) were inoculated with suspensions of M. oryzae conidia prepared in 0.25% gelatin at a concentration of 5×10^4^ conidia ml−1 using an artist's airbrush with high-pressure air. Rice leaves were sampled at 24, 48, and 72 hpi. The relative expression levels of PR1a, PR4, PR5, and PR10a in the infected rice was compared using quantitative RT-PCR. Normalization of average threshold cycle (Ct) was performed with that of O. sativa elongation factor 1α gene. Primers were synthesized according to Hao et al. (Plant Physiol Biochem. 2012; 60:150–156). (TIF) [file pone.0120627.s005.tif]
