# Supplementary material for: An S-(Hydroxymethyl)Glutathione Dehydrogenase Is Involved in Conidiation and Full Virulence in the Rice Blast Fungus Magnaporthe oryzae
Source: PLoS One. 2015 Mar 20;10(3):e0120627. doi: 10.1371/journal.pone.0120627 (PMC4368689; doi:10.1371/journal.pone.0120627)
Supplement: S1 Table — (PDF) [file pone.0120627.s006.pdf]

Table. S1 Primers used in this study

Primers	Sequence: 5'-3'	Comments
MG121	T*CCACCATGTTGG*TTCTCGAATTGTATACCT	pKO-MoGNO1 construction
MG122	T*GAATTC*GTTATTGAGGACAATTACGGT	pKO-MoGNO1 construction
MG123	A*TCTAGA*CTAGCTTACGGGAGTACAAG	pKO-MoGNO1 construction
MG124	ATG*GTCGAC*TGGCATCCATCGCCTTGT	pKO-MoGNO1 construction
MG125	GC*AAGCTT*AACACAATGTCTACAGTCGGAAAGACC	pYES2-MoGNO1 construction
MG126	AT*TCTAGA*CTTAGACGGCCCTCATGTC	pYES2-MoGNO1 construction
MG127	T*GAATTC*GGTTCTCGAATTGTATACCT	pBAR-MoGNO1R construction
MG128	GC*TCTAGA*GTCTGCGATTGACCCGTAATCC	pBAR-MoGNO1R construction
MG129	ATCCAGCCTTGCCGTCTTCG	Probe amplification
MG130	TCTCCGCCCTCCGCCTCTAA	Probe amplification
GNO1-F3	TGTCGTTGCTCTTTACACCC	MoGNO1 quantitative RT-PCR
GNO1-R3	CCCTTGCACTTGAACCTTG	MoGNO1 quantitative RT-PCR
Tub-F1	GGCCAATGCGGCAACCAA	MGG_00604 quantitative RT-PCR
Tub-R1	AGGACAGCACGGGGAACA	MGG_00604 quantitative RT-PCR

All primers were synthesized by Shanghai Sangon Biotechnology.
